# Isthmin inhibits glioma growth through antiangiogenesis in vivo

**DOI:** 10.1007/s11060-012-0910-8

**Published:** 2012-07-07

**Authors:** Bangqing Yuan, Ronghua Xian, Jianfang Ma, Yujian Chen, Chuangan Lin, Yaoming Song

**Affiliations:** 1 Department of Neurosurgery, The 476th Hospital of Fuzhou General Hospital, Fuzhou, 350025 Fujian China; 2 Department of Cardiovascular Diseases, Xinqiao Hospital, Third Military Medical University, Chongqing, 400037 China

**Keywords:** Isthmin, Glioma, Growth, Antiangiogenesis

## Abstract

Among glioma treatment strategies, antiangiogenesis emerges as a meaningful and feasible treatment approach for inducing long-term survival. Isthmin is a gene highly expressed in the isthmus of the midbrain–hindbrain organizer in Xenopus, and has recently been identified as a novel angiogenesis inhibitor. However, the potential of isthmin on the glioma angiogenesis has not been well studied. In the present study, we demonstrated that the recombinant adenovirus isthmin (Ad-isthmin) could inhibit VEGF-stimulated endothelial cell proliferation and induce apoptosis through a caspase-dependent pathway. In addition, Ad-isthmin significantly suppressed glioma growth through antiangiogenesis without apparent side effects. Taken together, our results demonstrated that isthmin could act as a novel angiogenesis inhibitor and might be utilized in the glioma antiangiogenesis therapy.

## Introduction

Malignant gliomas are among the most lethal tumors with a very dismal prognosis, despite advances in standard therapy, including surgery, radiation, and chemotherapy [1–3]. In an attempt to develop new therapeutic strategies and identify the molecular mechanism involved in glioma growth and progression, there has been extraordinary scientific interest in angiogenic responses associated with gliomas [4–6].

Angiogenesis plays an essential role in embryogenesis, but postnatal angiogenesis is limited to sites of abnormal vascular surface [7, 8]. An activated vascular endothelium can be induced by tissue injury or wound healing [9–11]. The process of postnatal angiogenesis is regulated by a continuous interplay of stimulators and inhibitors of angiogenesis, and their imbalance contributes to numerous inflammatory, malignant, ischemic, and immune disorders [12–14].

Isthmin, firstly discovered as a secreted protein highly expressed in the isthmus of the brain, was demonstrated as a novel endogenous angiogenesis inhibitor [15, 16]. Therefore, the finding provided the theoretic foundation for the glioma antiangiogenesis therapy. In this experiment, we constructed a recombinant adenovirus isthmin (Ad-isthmin), and explored the potential of glioma antiangiogenesis.

## Materials and methods

### Cell culture

The human glioma cell line U251 was purchased from ATCC (American Type Culture Collection). Cells were grown in RPMI 1640 supplemented with 10 % fetal calf serum, 1 × 10^5^ IU/L penicillin, 100 mg/L streptomycin, and 2-mmol/L glutamine in the presence of 5 % CO_2_. Human umbilical vein endothelial cells (HUVECs) were cultured on type-I collagen-coated dishes in endothelial basal medium containing endothelial cell growth supplements and 10 % FCS.

### Animals

All the experiments were carried out using 8-week-old female nude mice purchased from the Experimental Animal Center of Chinese Academy of Medical Science (Beijing, China). All animals were housed under specific pathogen-free conditions. Mice were allowed to acclimatize for at least 1 week before experiments commenced. All experimental procedures were carried out following approval of the Institutional Animal Care Committee.

### Construction of Ad-isthmin

Ad-isthmin was constructed using the Adeno-XTM Expression System (Clontech, Palo Alto, CA, USA) according to the manufacturer’s instructions. Briefly, the murine isthmin cDNA was cloned into the shuttle vector pDC315 and sequenced. The desired replication-deficient adenovirus containing the full-length cDNA of isthmin was generated by homologous recombination through co-transfection of plasmids pDC315-isthmin and pBHG1oXE1, 3Cre in HEK 293 cells using the DOTAP liposome reagent (Roche, Mannheim, Germany). After several rounds of plaque purification, isthmin adenovirus was amplified and purified from cell lysates by anding twice in CsCl density gradients. Viral products were desalted and stored at −80 °C in PBS containing 10 % glycerol (v. v). The infectious titer was determined by a standard plaque assay. A second recombinant, E1, E3-deleted adenovirus carrying the LacZ protein under the control of CMV promoter (Ad-LacZ), was used as a control vector.

### Ad-isthmin transduction assay

Transduction of U251 cells with Ad-isthmin was done in 6-well plates with 1 × 10^6^ U251 cells/well in 3 mL RPMI-1640 medium containing 10 % FBS. Virus was added to the wells at an MOI of 200 and the U251 cells were harvested after 24 h of incubation.

### Western blot analysis

Cells were harvested and lysed in a buffer containing 10 mM Tris–HCl, pH 7, 0.2 % Triton X-100 and protease inhibitors (Complete Mini EDTA-free; Roche Applied Science). Total protein (20 μg) was separated on 4–20 % SDS-PAGE and transferred to an immunoblot polyvinylidene difluoride (PVDF) membrane (Bio-Rad). After blocking the membrane in 3 % skim milk powder/PBS, it was probed with primary isthmin antibody (Santa Cruz Biotechnology, Santa Cruz, CA, USA), and then detected with a 1: 2,500 dilution of an HRP-conjugated goat anti-mouse IgG or a 1:2,500 dilution HRP-conjugated rabbit anti-goat IgG. After washing, a chemiluminescent substrate (Super Signal West dura extended; Pierce) was added to the membrane, which was then exposed to the ECL Hyperfilm (GE Healthcare; Amersham Biosciences).

### Cell proliferation inhibition assay

Cell proliferation was measured by a colorimetric assay using MTT. In brief, the HUVECs or U251 cells were seeded in 96-well plates in triplicate at 5 × 10^3^ cells/well and incubated in culture medium overnight. Then, the cells were treated with Ad-isthmin (1 × 10^9^ pfu) or controls in a total volume of 0.2 mL each well for 24 h. Thereafter, 20 μL of the indicator dye MTT solution (5 mg/mL) was added to each well and cultures were continued for 48 h at 37 °C and 5 % CO_2_. After centrifugation, the supernatant was removed from each well. The colored formazan crystal produced from MTT was dissolved with 0.15 mL DMSO, and the optical density (OD) value A490 was measured by the multiscanner autoreader (Dynatech MR 5000; Dynatech Laboratories, Chantilly, VA, USA). The following formula was used: cell proliferation inhibited (%) = [1 − (OD of the experimental samples/OD of the control) × 100 %]. In addition, after U251 cells were treated with Ad-isthmin (1 × 10^9^ pfu) or controls for 24 h, the supernatants were harvested and cocultured with HUVECs with the similar method. The cell proliferation inhibition was also calculated.

### Flow cytometric analysis of apoptosis

An annexin V-fluorescein isothiocyanate (FITC) apoptosis detection kit (Oncogene Research Products, Boston, MA, USA) was used to detect apoptosis. HUVECs or U251 cells (3 × 10^5^ per well) were cultured in coated 6-well plates in complete RPMI 1640 medium overnight at 37 °C. Ad-isthmin (1 × 10^9^ pfu) and VEGF (15 ng/mL) (Calbiochem, UK) were added to the culture medium, and incubated for 48 h prior to apoptosis detection. Then, the cells were harvested, washed with ice-cold PBS twice, and resuspended in binding buffer (10 mM of Hepes, pH 7.4, 1 50 Mm of NaCL, 2.5 mM of CaCL_2_, 1 mM of MgCL_2_, 4 % bovine serum albumin). Annexin V-fluorescein isothiocyanate (0.5 mg/mL) and propidium iodide (0.6 mg/mL) were then added to a 250-mL aliquot (1 × 10^6^ cells) of this cell suspension according to the protocol of the manufacturer. After 15 min incubation in the dark at room temperature, stained cells were immediately analyzed on a flow cytometer (Beckman Coulter, USA). All the samples were assayed in triplicate, and the cell apoptosis rate calculated using the following formula: apoptosis rate = (apoptotic cell number/total cell number) × 100 %.

### Measurement of caspase-3 activity

The activity of caspase was determined using caspase colorimetric assay kit (Sigma, USA), according to the manufacturer’s protocol. Briefly, the treated and control cells were washed with ice-cold PBS and lysed in a lysis buffer. The cell lysate was tested for protease activity using a caspase-specific peptide, conjugated to the color reporter molecule *p*-nitroanaline. The chromophore *p*-nitroanaline, cleaved by caspases, was quantitated with a spectrophotometer at a wavelength of 405 nm. The caspase enzymatic activities in cell lysate were directly proportional to the color reaction.

### Tumor xenografts in nude mice

Eight-week-old nude mice were challenged with subcutaneous (s.c) injection of 1 × 10^6^ U251 glioma cells into the right flank to induce primary tumors. Seven days after tumor cell inoculation, mice were divided randomly into three groups (10 mice per group) and were received an intratumor injection of Ad-isthmin (1 × 10^9^ pfu) or Ad-LacZ (1 × 10^9^ pfu). The control mice received 100 μL PBS. Tumor volume of the mice was measured using a caliper. Tumor volume was measured in two dimensions and calculated as follows: length/2 × width^2^.

### Orthotopic glioma model in nude mice

Eight-week-old nude mice were anesthetized, and a 1-mm burrhole was drilled into the skull 3.5 mm lateral to the bregma. Then, 1 × 10^6^ U251 glioma cells were injected through the screw into the basal ganglia of the mice. One day after tumor cell inoculation, mice were divided randomly into three groups (10 mice per group) and were received an intracerebral delivery of Ad-isthmin (1 × 10^9^ pfu) or Ad-LacZ (1 × 10^9^ pfu). The control mice received 5 μL PBS. Then, 15 days after the tumor challenge, the mice were killed. Mouse brains were removed from the cranial cavity, fixed in formalin overnight, bisected coronally, and embedded in paraffin. Serial sections (5 μm thick) were stained with hematoxylin-eosin. The maximum cross-sectional area of the intracranial glioblastoma xenografts was determined by computer-assisted image analysis with a Leica Quantimet 500 (Leica, Hamburg, Germany). Tumor volume was estimated according to the following formula: volume = (square root of maximal tumor cross-sectional area)^3^. In addition, the mean lifespan of the mice were also observed.

### Microvessel density detection

Microvessel density (MVD) has been shown to correlate with angiogenic activity and tumor progression. Then, 21 days after the tumor challenge, the mice were killed. The cryosections of glioma were prepared after freezing the removed tumor tissue in Tissue-Tek (Sakura Finetech) using methylbutanol and liquid nitrogen. Thereafter, cryosections of 5 μm were cut and fixed in acetone. Sections were blocked in 3 % BSA/PBS and incubated with 1 μg/mL rat anti-mouse CD31 (PECAM-1, BD Biosciences). The sections were then stained with labeled streptavidin–biotin reagents. Microvessel density (MVD; diameter <50 μm) was determined by counting ten randomly chosen high-power fields (HPF ×200) from each tumor.

### Evaluation of side-effects

Seven days after injection, a full thickness wound was excised from the dorsum of the mice. The defect was created by elevating the skin and panniculus carnosus in the center of the outlined defect using forceps, followed by excision of the outlined area using scissors. Wound area was measured twice weekly. Fifteen days after this excision, mice were killed and scar tissues were removed for histological examination. We evaluated vaccinated and control mice by both the wire hang test and the footprint test, as well as by overall behavior and determination of body weight. To test hematopoiesis, animals were subjected to complete peripheral blood counts.

### Statistical analysis

The statistical significance of differential findings between experimental groups and controls was determined by Student’s *t* test and considered significant if two-tailed *P* values were < 0.05.

## Results

### Ad-isthmin transduction and detection

**Fig. 1:**
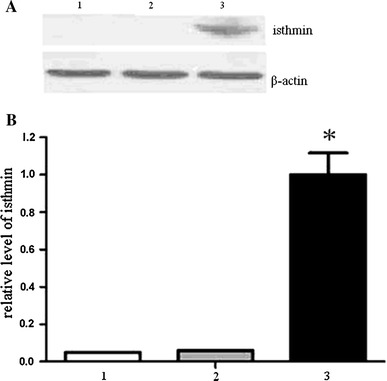
**a** The expression of isthmin in U251 cells was analyzed by western blot analysis. U251 cells were transduced with Ad-isthmin or Ad-LacZ at an MOI of 200 for 24 h. The expression of isthmin increased significantly after Ad-isthmin transfection. However, the expression of isthmin after Ad-LacZ transduction and non-treated U251 cells could not be detected. **b** The expression of isthmin was analyzed by semi-quantitative western blot using β-actin for standardization. *1* non-treated U251 cells, *2* U251 cells transduced with Ad-LacZ, *3* U251 cells transduced with Ad-isthmin

To identify whether Ad-isthmin could transduce into cells and mediate the gene expression, we detected the protein by western blot assay. In accordance with protocols mentioned above, U251 cells were transduced with Ad-isthmin or Ad-LacZ at MOI 200 for 24 h. As Fig. 1a demonstrates, the expression of isthmin was detected after Ad-isthmin transduction. However, isthmin could not be detected after Ad-LacZ transduction and non-treated U251 cells.

### Ad-isthmin inhibits proliferation of HUVECs

**Fig. 2:**
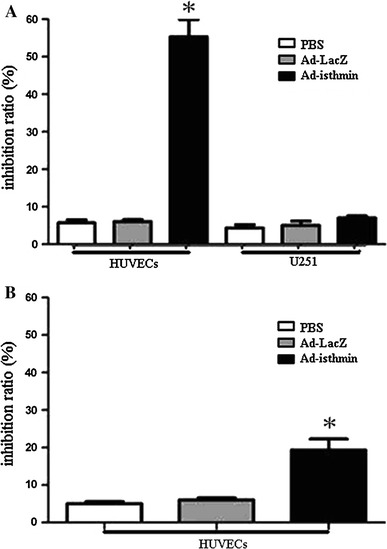
Inhibitory effect of Ad-Isthmin on cell proliferation. **a** HUVECs or U251 cells were seeded in 96-well plates at 5 × 10^3^ cells/well and incubated in culture medium overnight. The cells were transduced with Ad-isthmin (1 × 10^9^ pfu) or controls for 24 h. **b** U251 cells were seeded in 96-well plates at 5 × 10^3^ cells/well and incubated in culture medium overnight. The cells were transduced with Ad-isthmin (1 × 10^9^ pfu) or controls for 24 h. The supernatant were harvested and cocultured with HUVECs with the similar method. Then MTT solution (5 mg/mL) was added and cultured for 48 h. The colored formazan crystal produced from MTT was dissolved with 0.15 mL DMSO then the optical density (OD) value A490 was measured. The data are the averages from three independent triplicate experiments. *P < 0.05 compared with control groups

To determine whether Ad-isthmin could inhibit VEGF-stimulated proliferation, we detected the metabolic activity at 48 h by the MTT assay. As Fig. 2a demonstrates, the HUVEC viability was reduced significantly after transduced with Ad-isthmin compared with control groups. The HUVEC proliferation inhibition rate reached 58.4 ± 7.2 %. However, these changes in the U251 cell groups were not significant.

### Flow cytometry for analysis of apoptotic cells

**Fig. 3:**
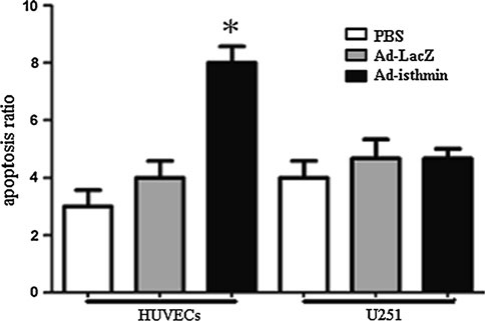
Ad-isthmin induces cell apoptosis in vitro. 2 days after transduction of Ad-isthmin, Ad-LacZ or PBS, Annexin V-fluoresceinisothiocyanate (0.5 mg/mL) and propidium iodide (0.5 mg/mL) were then added to a 250-mL aliquot (5 × 10^6^ cells) of this cell suspension. After 15 min incubation in the dark at room temperature, stained cells were immediately analyzed by Flow Cytometry (Coulter Biosciences). Apoptosis cells were determined by AnnexinV-positive and propidium iodide (PI)-negative cells. The data are the averages from three independent triplicate experiments. *P < 0.05 compared with control groups

We hypothesized that the Ad-isthmin inhibited HUVEC proliferation by inducing cell apoptosis. In order to explore this possibility, we measured the apoptosis levels of transduced HUVECs. As Fig. 3 demonstrates, the results showed that HUVEC apoptosis rates increased significantly compared with control groups. However, these changes in the U251 cell groups were not significant.

### Ad-isthmin activated the apoptosis through caspase-3 pathway

**Fig. 4:**
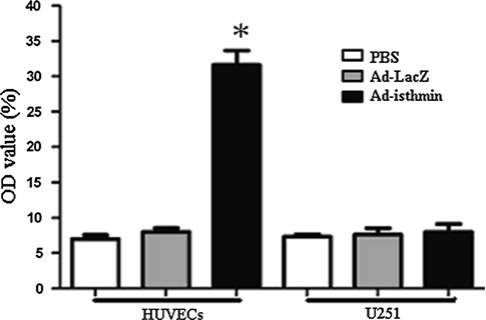
Analysis of caspase-3 activities. The variation of caspase-3 activities in HUVECs was measured using caspase colorimetric assay kit. After the HUVECs were transduced with Ad-isthmin, Ad-LacZ, or PBS, the cell lysates were tested for protease activity using a caspase-specific peptide, conjugated to the color reporter molecule *p*-nitroanaline. The chromophore *p*-nitroanaline, cleaved by caspases, was quantitated with a spectrophotometer at a wavelength of 405 nm. The caspase enzymatic activities in cell lysate were directly proportional to the color reaction. The data are the averages from three independent triplicate experiments. *P < 0.05 compared with control groups

To determine whether Ad-isthmin induced apoptosis through a caspase-dependent pathway, we further investigated caspase-specific activities after Ad-isthmin transduced HUVECs. As shown in Fig. 4, Ad-isthmin remarkably increased the caspase-3 activities compared with control groups.

### Ad-isthmin inhibited the tumorigenicity of U251

**Fig. 5:**
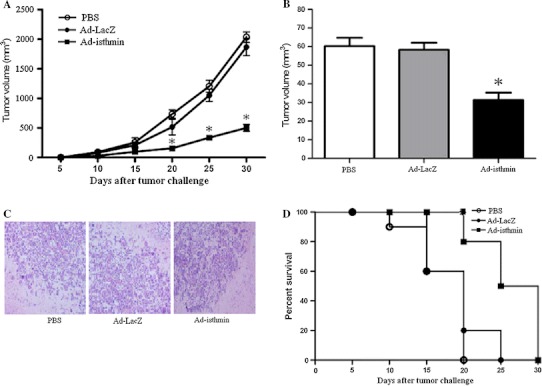
Ad-isthmin inhibits tumor growth of nude mice. **a** Eight-week-old nude mice were challenged with subcutaneous (s.c) injection of 1 × 10^5^ U251 glioma cells into the right flank to induce primary tumors. Seven days after tumor cell inoculation, mice were divided randomly and were received an intratumor injection of Ad-isthmin (1 × 10^9^ pfu), Ad-LacZ (1 × 10^9^ pfu), or 100 μL PBS (10 mice per group). Tumor volume was measured in two dimensions and calculated as follows: length/2 × width^2^. **b** Eight-week-old nude mice were challenged with intracerebral injection of 1 × 10^6^ U251 glioma cells into the basal ganglia of the mice. One day after tumor cell inoculation, mice were divided randomly and were received an intracerebral delivery of Ad-isthmin (1 × 10^9^ pfu), Ad-LacZ (1 × 10^9^ pfu), or 5 μL PBS (10 mice per group). Fifteen days after the tumor challenge, the mice were killed. Mouse brains were removed, fixed and embedded. Serial sections (5 μm thick) were stained with hematoxylin-eosin (HE). Tumor volume was estimated as follows: (square root of maximal tumor cross-sectional area)^3^. **c** Representative HE staining section of mouse brain after intracerebral injection of U251 glioma cells. **d** Percent survival of nude mice challenged with intracerebral injection of 1 × 10^6^ U251 glioma cells (10 mice per group). The data are the averages from three independent triplicate experiments

The effect of Ad-isthmin on tumorigenicity of U251 cells was evaluated following subcutaneous or intracerebral injection of 1 × 10^6^ cells into nude mice for each group and the tumor growth was monitored. As shown in Fig. 5a, the subcutaneous tumor volume expanded rapidly after 15 days of tumor challenge in the control groups. However, in Ad-isthmin group, the tumor volume expanded steadily (*P* < 0.05). In addition, as shown in Fig. 5b, the orthotopic tumor volume at the end of the experiment revealed a statistically significant reduction of tumor growth on treatment with Ad-isthmin as compared with control groups. And Fig. 5d demonstrates that Ad-isthmin treatment resulted in a significant increase in survival time (*P* < 0.05). These results indicated that Ad-isthmin exerted a strong growth suppressive effect on U251 cells in vivo.

### Ad-isthmin inhibited tumor angiogenesis

To assess whether Ad-isthmin could inhibit tumor angiogenesis, angiogenesis of tumor tissue was evaluated by counting the number of microvessels on the sections stained with anti-CD31 antibody.

**Fig. 6:**
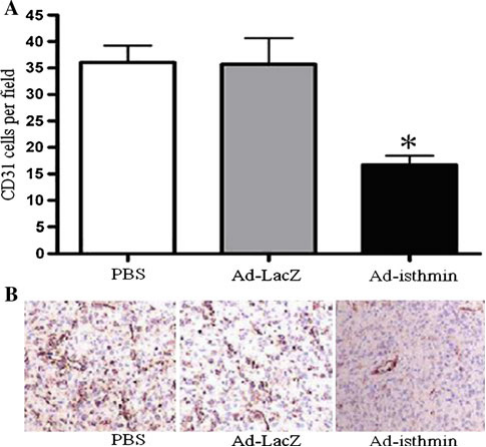
Inhibition of angiogenesis in the tumor tissue. 21 days after the tumor challenge, the mice were sacrificed, and the tumor tissues were fixed in acetone, and stained with an antibody reactive to CD31 as described for microvessel density (MVD) analysis. **a** Vessel density was determined by counting the number of the microvessels per high-power field in tumor sections stained with antibody reactive to CD31. **b** The representative immuno histochemical analysis of CD31 expression in the tumor of Ad-isthmin and control groups. The data are the averages from three independent triplicate experiments. *P < 0.05 compared with control groups

As shown in Fig. 6, the average number and vessels per high-power field were both lower in the Ad-isthmin group compared with control groups. The results suggested Ad-isthmin could potentially inhibit tumor angiogenesis.

### Analysis of the side-effects of Ad-isthmin

To further identify whether Ad-isthmin had an effect on normal physiological angiogenesis, we analyzed wound healing time using a cutaneous excision wound model. A full thickness wound was created on the dorsum of immunized and control mice 10 days after Ad-isthmin adminstration. Wound areas were measured until completely healed. The results demonstrated that no significant difference in wound healing was observed between experimental and control mice. Furthermore, neuromuscular performance, as determined by both wire hang and footprint tests, body weight, overall behavior, balancing tests, and peripheral blood examination, did not indicate any impairment (data not shown).

## Discussion

Malignant gliomas, the most common subtype of primary brain tumor, are aggressive, highly invasive, and neurologically destructive [17–19]. First-line treatment of gliomas consists of surgery and radiotherapy, followed by chemotherapy with temozolomide [20–22]. However, even with this strong regimen, the prognosis of patients with the most malignant variant, glioblastoma multiforme, is poor [23–25]. Because of the lack of effective treatments and the high vascularity that characterizes these tumors, antiangiogenic therapy of gliomas is being studied. This approach is supported by encouraging preclinical data in both in vitro and in vivo models [26–28]. Clinical studies have shown that these agents do not cause high toxicity; and due to the effect they exert on vessel permeability, patients can avoid the use of corticosteroids and their accompanying adverse effects [29–31]. Antiangiogenic therapy is a promising approach for the treatment of cancer. In practice, however, only a subset of patients who receive antiangiogenic drugs such as bevacizumab demonstrate a significant response. A key challenge, therefore, is to discover novel moleculars that are predictive of response to antiangiogenic therapy.

Isthmin is a secreted 60-kD protein containing a thrombospondin type 1 repeat domain in the central region and an adhesion-associated domain in MUC4 [32]. Isthmin was first identified in Xenopus but its function is not yet known. During the neuronal stage, isthmin is highly expressed in the isthmus organizer, the signaling center located at the midbrain–hindbrain boundary (MHB). Additional expression was detected in the paraxial mesoderm and neural folds in the tailbud stage as well as in notochord in the neuronal stage [33]. In a recent study, it was demonstrated that isthmin was a novel angiogenesis inhibitor and inhibited endothelial cell (EC) capillary network formation [16]. Therefore, to explore the potential of isthmin in glioma antiangiogenesis, we constructed the adenovirus isthmin and studied the effect.

To determine whether Ad-isthmin could inhibit VEGF-stimulated proliferation of HUVECs, we detected the metabolic activity by the MTT assay. The results demonstrated that the cell viability was reduced significantly after transduced with Ad-isthmin compared with control groups. The data suggested isthmin could inhibit proliferation of HUVECs through inhibiting VEGF. To further explore the possibility, we also analyzed the apoptotic cells and found that Ad-isthmin activated the HUVEC apoptosis through the caspase-3 pathway. In addition, we evaluated the effect of Ad-isthmin on the tumorigenicity of U251 following subcutaneous or intracerebral injection, and found that Ad-isthmin exerted a strong growth suppressive effect on U251 cells through inhibiting tumor angiogenesis in vivo. Furthermore, Ad-isthmin could improve the mean survival of tumor-bearing mice. Lastly, antiangiogenic agents have some side effects profile, likely due to inhibition of normal physiologic angiogenesis. To identify the side effects and provide data for safety testing, we analyzed wound healing time, neuromuscular performance, body weight, overall behavior, balancing tests, and peripheral blood counts. The results showed there were no significant differences between the Ad-isthmin and control groups.

Among antiangiogenic agents, thromboembolic and haemorrohage events have been reported. This side effect has been related to the antiangiogenic effect of this immunomodulator [34, 35]. Some efficacy has been reported with the use of aspirin, oral anticoagulant, or low molecular weight heparin. From published data, full dose oral anticoagulants appear to confer the highest hemorrhagic risk and perhaps low molecular weight heparin the best benefit–risk ratio. Moreover, while treatment of other tumor types may be improved by combining chemotherapy with anti-angiogenic drugs, inhibiting angiogenesis in GBM may antagonize the efficacy of chemotherapeutic drugs by normalizing the blood–brain barrier function.

Together, we have provided the evidence that Ad-isthmin can inhibit VEGF-stimulated endothelial cell proliferation and significantly suppress glioma growth through antiangiogenesis without apparent side effects. The findings might be valuable as a potential strategy for glioma antiangiogenesis therapy in the future.
